# Dr. Clayton A. Button

**Published:** 1915-06

**Authors:** 


**Dr. Clayton A. Button,** Buffalo 1898, died at his home in Holland, Feb. 24, aged 62.

